# Hand injuries in sports – a retrospective analysis of 364 cases

**DOI:** 10.1186/s12891-020-03807-z

**Published:** 2020-12-08

**Authors:** Viola A. Stögner, Alexander Kaltenborn, Hans Laser, Peter M. Vogt

**Affiliations:** 1 Department of Plastic, Aesthetic, Hand and Reconstructive Surgery, Hannover Medical School, Carl-Neuberg-Strasse 1, 30625 Hannover, Germany; 2 Department of Trauma and Orthopedic Surgery, Plastic, Hand and Reconstructive Surgery, Armed Forces Hospital Westerstede, Lange Strasse 38, 26655 Westerstede, Germany; 3 Department for Educational and Scientific IT Systems, Centre for Information Management, Hannover Medical School, Carl-Neuberg-Strasse 1, 30625 Hannover, Germany

**Keywords:** Sports-related, Injury, Injuries, Trauma, Hand, Equestrian sports

## Abstract

**Background:**

Hand injuries are common in sports and associated with high dropout rates and costs.

Hence, efforts should strive for further risk prevention measures in order to increase safety in sports. This implies knowledge of sports injury risk profiles. So far, major surveillance programs exist mainly in Anglo-American countries, reflecting the specific concerns of sports in this part of the world. Data on sports injuries within Europe are scarce. As sports behaviour appears to vary demographically, we hypothesised that risk injury profiles differ as well.

**Methods:**

To assess whether the described sports injuries of the hand are applicable to the German population, we performed a five-year retrospective, single-centre analysis of sports-related hand injuries, using data from the Enterprise Clinical Research Data Warehouse of the Hannover Medical School.

**Results:**

Notable differences in comparison to other data were observed. Ball sports, cycling and equestrian sports caused most of the recorded hand injuries, which were predominantly fractures of the wrist and hand. Hand injuries in equestrian sports were associated with significantly higher operation and hospitalisation rates as well as a significantly longer inpatient treatment.

**Conclusion:**

Risk profiles for sports-related hand injuries appear to differ not only in terms of age- and sex, but also geographically. Nation- and Europe-wide hand trauma registries as well as a broad registry participation are necessary in order to accurately assess the risk patterns in Europe; henceforth reducing hand injuries and their sequelae.

**Supplementary Information:**

The online version contains supplementary material available at 10.1186/s12891-020-03807-z.

## Background

The benefits of sports to physical and mental health are globally evident and recognised [1]. Regular physical activity improves social skill development and self-esteem in children while contributing strongly to psychological health and stress-compensation in adults, and is therefore associated with enhanced quality of life in young and old [2–5].

However, the omnipresent risk of injury is still the major drawback for every athlete, in both professional and amateur sports [6]. Previous prevention measures, such as rule changes or the introduction of protective clothing, managed to increase safety in many sports [7–9]. Rule changes made by the Amateur International Boxing Association (AIBA) in 2013 for example, resulted in a substantial decrease of boxing-related upper extremity injuries between 2012 and 2016, shown most impressively in a decline of hand injuries by 33% [7, 10]. Nevertheless, sports injuries still occur with high incidences, showing varying risk profiles within the different types of sport [11, 12]. Injuries of the hand account for approximately 25% of all sports-related injuries [13–22]. Therefore, the hand is at high risk for injury during sports activities. Within the National Football League Scouting Combine from 2009 to 2015, the hand, with 33.5%, was among the top five of the affected body regions [22]. The dimension of direct and indirect costs of hand and wrist injuries goes along with a great economic burden [23–25]. So far, evidence of risk profiles in sports is mainly based on data from large surveillance programs of the Anglo-American region, including the USA [18, 22, 26], Canada [27] and Australia [28–30]. In order to investigate the most common sports injuries of the hand and wrist [21, 31] in a European study population and to contribute to the establishment of sports injury risk profiles, the following epidemiological study, based on data from a major hand trauma centre in northern Germany, was performed.

## Methods

Sports-related hand injuries, treated at the Department of Plastic, Aesthetic, Hand and Reconstructive Surgery of the Hannover Medical School, a level I trauma centre as well as FESSH (Federation of European Societies for Surgery of the Hand)-accredited Hand Trauma Centre, were reviewed retrospectively from February 2013 to February 2018. Patients with a verified sports injury of the hand were included regardless of their sex or age. The study was approved by the Hannover Medical School’s Ethics Committee.

### Variables

Due to the small number of cases in the individual sport groups, sports were assigned to different sport supergroups for further statistical analyses. Since there is no evidence-based sports classification available, sport supergroups were defined in the most reasonable way (ball sports played with an assistive device such as a bat, racquet or stick – collectively referred to as” ball sports with a bat”, ball sports played without a bat, racquet or stick – collectively referred to as “ball sports without a bat”, “gymnastics”, “martial arts”, “climbing”, “outdoor sports”, “precision sports”, “cycling”, “equestrian sports”, “skating”, “water sports”, “winter sports” and “miscellaneous”) (Table 2). Equestrian sports injuries were defined as such that occurred during horse riding itself, as well as the horse handling in course of equitation, such as leading the horse by the rein or bridling a horse.

All included cases were reviewed for the following variables: age (continuous), sex (categorical), sport and sport group (categorical), treatment type (categorical), hospitalisations (categorical), length of hospital stay (LOS) (continuous) as well as ICD-10-GM codes (categorial).

### Data acquisition

**Fig. 1:**
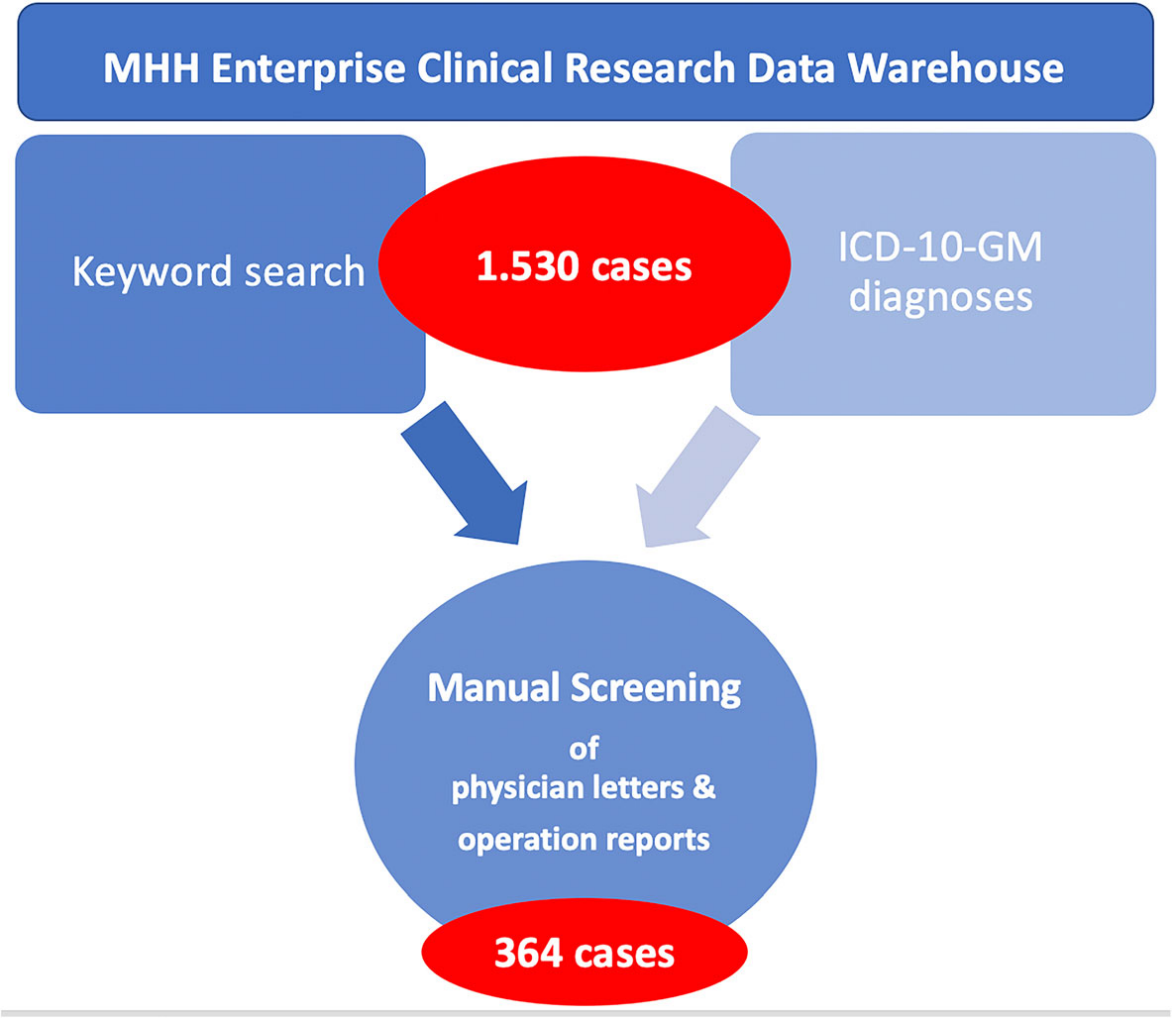
Consort flow diagram depicting the process of data acquisition

Physician discharge letters and operation reports of the above named department were extracted by the Enterprise Clinical Research Data Warehouse (ECRDW) of the Hannover Medical School, which includes data regarding clinical routine (such as diagnoses, laboratory findings, text findings) of > 2.3 million patients [32]. The relevant cohorts were identified using a specific keyword search (Additional file 1) as well as the ICD-10-GM codes (International Statistical Classification of Diseases and Related Health Problems 10th Revision German Modification) for hand injuries (Table 1). A keyword list with 147 different relevant terms (named entities) was used to identify equivalent entities in the medical documents (named entity recognition approach) [33], which resulted in a total of 1530 cases. All collected data were automatically exported from the ECRDW into Microsoft Excel spreadsheets. The medical reports of these cases have been extracted for further processing. Since ICD-10-GM codes and the keyword search were not sensitive enough to verify that hand injuries were actually sports-related, additionally manual screening for those indicators of each of the extracted medical reports was performed. Finally, 364 patients with an ICD-10-GM diagnosis for a hand injury and a sports-related injury aetiology could be included in the analysis (Fig. 1). By means of the unique patient identification number, we verified that no patient was counted more than once.

**Table 1:** ICD-10-GM codes used in the data extraction process

**S60.**	Superficial injury of the wrist and hand
**S61.**	Open wound of the wrist and hand
**S62.**	Fracture of the wrist and hand
**S63.**	Luxation, sprain and strain of joints and ligaments at height of the wrist and hand
**S64.**	Injury of nerves at height of the wrist and hand
**S65.**	Injury of blood vessels at height of the wrist and hand
**S66.**	Injury of muscles and tendons at height of the wrist and hand
**S67.**	Crushing injury of the wrist and hand
**S68.**	Traumatic amputation of the wrist and hand
**S69.**	Other not further specified injury of the wrist and hand

### Statistical analysis

For statistical analysis the IBM SPSS Statistics program (Version 26, released 2019) was used. To assess the distribution of continuous data a Kolmogorov-Smirnov was performed. For the comparison of non-parametrically distributed data, the Mann-Whitney-U test was applied. In case of parametric distribution, the data were compared with the Student’s two-sided t-Test. Categorical variables were analysed with the Pearson’s chi-squared test.

## Results

**Fig. 2:**
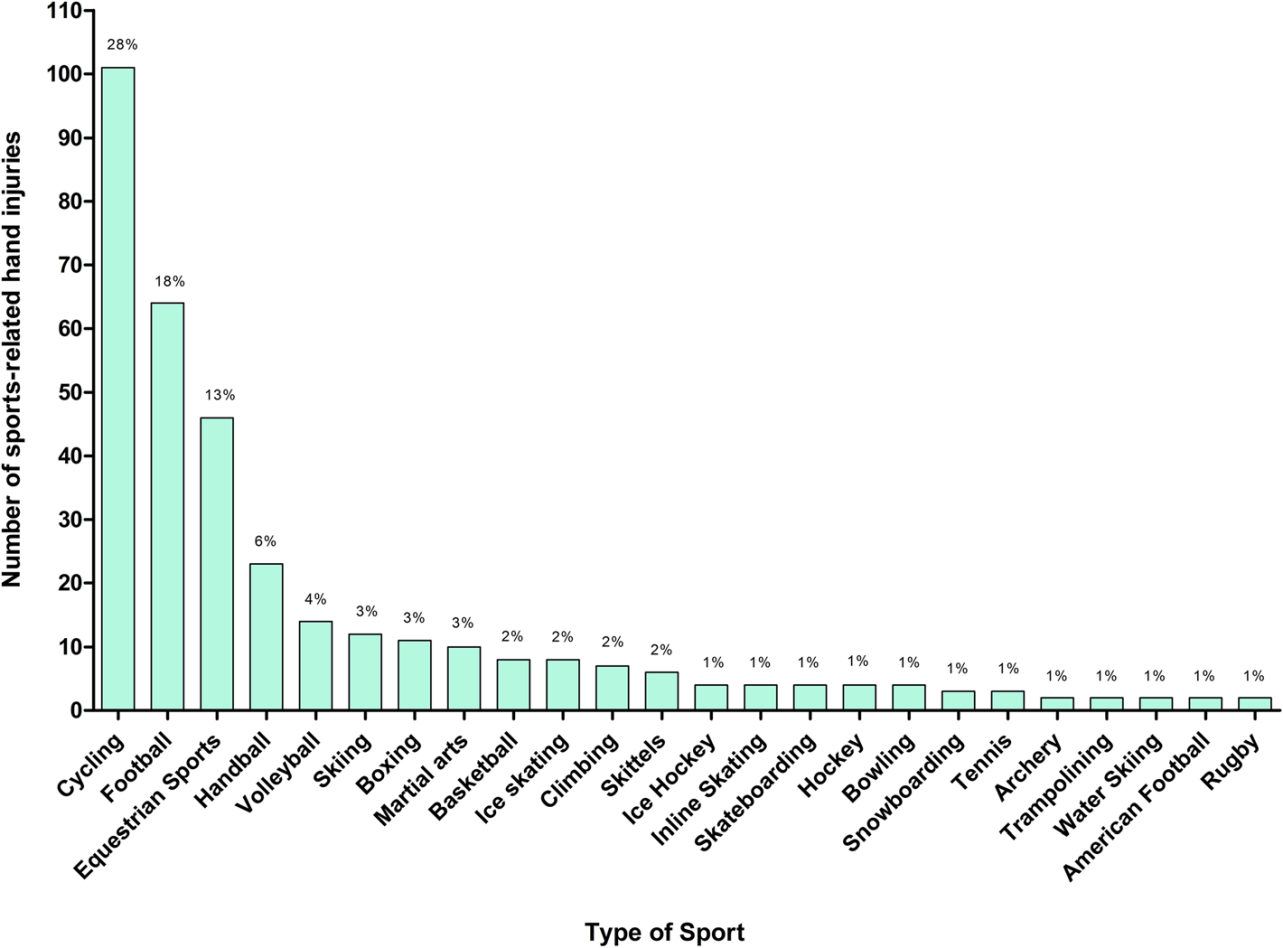
Absolute and relative numbers of hand injuries according to different types of sport. Only sports responsible for ≥1% of the recorded hand injuries were included in this figure

A total of 364 hand injuries, caused by 42 different types of sport, were treated (Fig. 2, Table 2).

**Table 2:** Absolute (n) and relative (%) numbers of patients with hand injuries and the associated type of sport and sports super group respectively

Sports group/sports	Numbers (%)
**Ball sports without a bat**	**114 (31%)**
Football	64
Handball	24
Volleyball	14
Basketball	8
Rugby	2
American Football	2
Ball sports (not further specified)	1
**Cycling**	**101 (28%)**
**Equestrian sports**	**46 (13%)**
**Winter sports**	**24 (7%)**
Skiing	12
Ice skating	8
Snowboarding	3
Tobogganing	1
**Martial arts**	**21 (6%)**
Boxing	11
Martial arts	10
**Ball sports with a bat**	**13 (4%)**
Hockey	4
Ice hockey	4
Tennis	3
Cricket	1
Squash	1
**Precision sports**	**11 (3%)**
Skittles	6
Bowling	4
Darts	1
**Skating**	**8 (2%)**
Skateboarding	4
Inline Skating	4
**Water sports**	**7 (2%)**
Water Skiing	2
Sailing	1
Surfing	1
Rowing	1
Canoe Polo	1
Swimming	1
**Climbing**	**7 (2%)**
**Gymnastics**	**5 (1%)**
Trampolining	2
Turning	1
Bench Press	1
Fitness Training	1
**Miscellaneous**	**4 (1%)**
Archery	2
Ballet Dancing	1
School sports (not further specified)	1
**Outdoor sports**	**3 (1%)**
Hiking	1
Fishing	1
Jogging	1
**Total**	**364 (100%)**

**Fig. 3:**
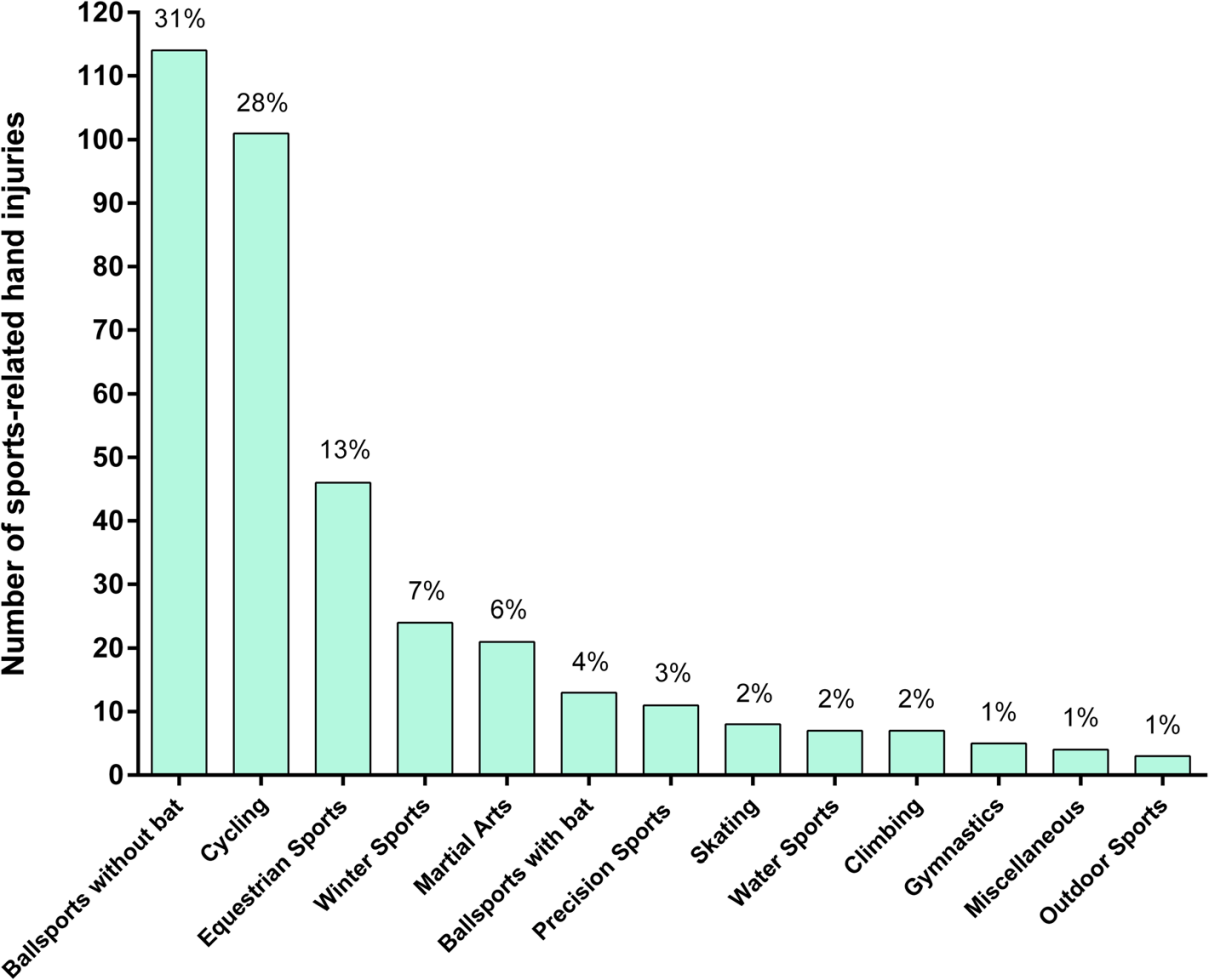
Absolute and relative numbers of hand injuries according to different sports groups

The sport leading to most of the hand injuries was cycling (*n* = 101, 28%), followed by football (*n* = 66, 18%) and equestrian sports (*n* = 46, 13%). According to supergroup categorisation the majority of sports-related hand injuries occurred in “ball sports without a bat” (*n* = 114, 31%), again followed by cycling (*n =* 101, 28%) and equestrian sports (*n =* 46, 13%) (Fig. 3).

### Sex

Overall, males were affected more often than females (females *n* = 118, 32%; males *n* = 246, 68%). Significant sex-dependent differences were observed in equestrian sports and football. 84% (*n* = 39) of the equestrian-related hand injuries affected the female study population (*p* < 0.001). With 33% equestrian sports accounted for most of the female sports injuries of the hand. Significantly more male patients suffered hand injuries during ball sports without a bat (*n* = 101, 89%; *p <* 0.001), which is mainly due to the large part of football-associated injuries. Statistical analysis revealed no significant difference between patients’ sex and LOS nor between sex and the need for operation respectively (*p* = 0.306; *p* = 0.703).

### Age

Half of the study population was aged between 19 and 45 years, with a mean patient age of 32 ± 17 years (range 3–89). There was no significant difference between the age of female and male patients (*p* = 0.343). Patient’s age was further not correlated with the necessity of an inpatient or operative treatment (*p* = 0.648; *p* = 0.556 respectively). However, the analysis revealed significant differences between patient’s age distribution and sports groups (*p* = 0.015). Ball sports without a bat, climbing and gymnastics affected significantly more young people (*p* < 0.001; *p =* 0.044; *p =* 0.015), while cycling-related hand injuries occurred significantly more often among the elderly study population (*p <* 0.001).

### Operation, Hospitalisation, LOS

218 (60%) patients received an operative treatment, while 166 patients (46%) were hospitalised due to sports-related hand injuries. 76% (*n* = 35) of all equestrian-related hand injuries were treated operatively (*p* = 0.017), while 63% (*n* = 29) of these injuries required hospitalisation (*p* = 0.011). The mean length of inpatient stay was 3.7 ± 4.47 days (range 1–27). Equestrian Sports led to a significantly longer LOS (Equestrian sports mean 3.2 ± 5.2 days vs. total study population mean 1.7 ± 3.5 days; *p* = 0.003). There was only one case where a patient was hospitalised without receiving surgical therapy, as the patient refused operation during the course of treatment.

### ICD-10-GM

**Fig. 4:**
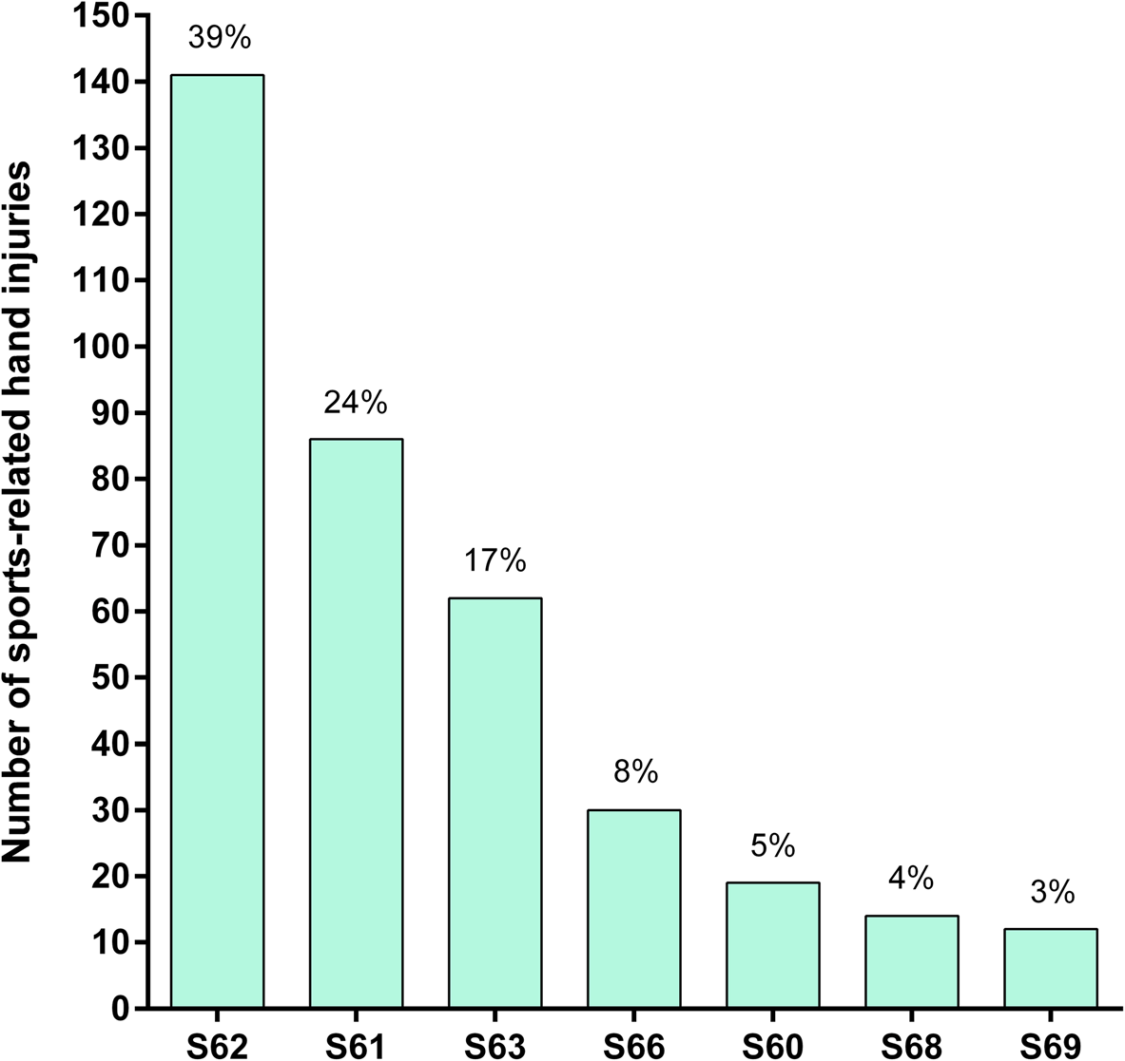
Absolute and relative numbers of sports-related hand injuries according to ICD-10-GM codes

According to the principal diagnoses codes, the most frequent injury type was “Fractures of the wrist and hand” S62.- (*n* = 141, 39%), followed by “Open wounds of the wrist and hand” S61.- (*n* = 86, 24%) and “Luxations, sprains and strains of joints and ligaments at the height of the wrist and hand” S63.- (*n* = 62, 17%) (Fig. 4).

There was no significant difference between patients’ sex and injury type (ICD-10-GM diagnosis) (*p* = 0.595). Fractures accounted for most hand injuries in ball sports without a bat (*p* = 0.007; *n* = 53, 47%) and equestrian sports (*n* = 15, 32.6%; *p* < 0.001). Among the cycling cohort there was a trend towards more S61.- (*n* = 34, 34%) and S62.- (*n* = 35, 35%) injuries. 64% (*n* = 9) of all amputation injuries of the hand and wrist (S68.-) occurred during equestrian sports, which is significantly more than in any other sports group (*p <* 0.001).

## Discussion

Besides the proven health benefits, the risk of injury represents the major drawback of sportive activities. Injury risk profiles may parallel demographic patterns, as well as social trends [1, 34–36]. Contrary to the United States, where a vast majority of sports-related hand injuries occur during American football, gymnastics, wrestling and basketball [34], we identified cycling, football and equestrian sports as the major causes of hand injuries in our cohort. Most of the observed injuries occurred in males and within the first two age quartiles, which coincides with with previous reports [4, 37]. Ball sports played without an assistive device, climbing and gymnastics were predominantly responsible for hand injuries in the younger population, while a shift towards more cycling-related hand injuries could be observed in the elderly population. Equitation-related hand injuries do not only represent one of the most common sports injuries in our analysis, but were also associated with a significantly higher operation and hospitalisation rate. The need for an operative treatment is mostly due to the increased rate of fractures in this sub-cohort. Furthermore, this sub-cohort accounted for nine out of 14 traumatic finger amputations in this study population. These findings support our clinical notion that complex injury patterns are common in equitation sports. Interestingly, equestrian sports seem to be underrepresented in the current literature. Therefore these findings might well add relevant information for clinical practice. The 2018 German examination regulations in equestrian sports specify mandatory gloves only in dressage and horse driving sports [38]. Regarding the high incidences of trauma and their severity, a broadening of this rule to all disciplines in equestrian sports, should be discussed.

According to previous studies, men represent the greater risk group for sports injuries. These observations can be confirmed by the presented results, where men are affected more than twice as often as women. Younger people, representing the physically most active population group, are at highest risk for sports injuries. 50% of the study population was aged between 19 and 45 years. Participation of young individuals in organised sports and high-frequency trainings are of increasing popularity, especially in the western world [2, 20, 39]. Simultaneously, a rising competitiveness, in terms of force and speed, is observable. Sport is one of the main causes of injuries in adolescents [40], with an increased risk for complicated injuries and long-term damage [39]. However, risk profiles appear to be dynamic, depending on demographic and social trends. Taking into account rising life expectancy and increasing sports activities throughout one's life in developed countries, an increased incidence of sports injuries among the elderly population has to be expected [34]. The dynamics of sports injury risk profiles raises the demand for injury prevention measures. This, however, requires knowledge of sport specific injury risks and injury patterns respectively.

An analysis of injuries occurring in German club sports from 1987 to 2012, without specification on the hand, showed high injury risks in ball sports such as football, handball, basketball or volleyball [41]. These data, however, did not include the non-organised leisure sports, such as cycling, which proves to be a major risk sport in our patient population. Further major epidemiologic and demographic surveys on sports injuries in Germany go back to the years 1986 and 1999, already indicating differences of injury-causing sport types between Germany and the USA. Previous national surveys, seeking to assess sports injuries in Germany, are rare and mainly based on questionnaires [42–44]. According to our knowledge, this is the first study analysing sports injuries specifically to the hand in a German study population.

A clear limitation of this study is its single-centre character. Although the Department of Plastic, Aesthetic, Hand and Reconstructive Surgery of the Hannover Medical School is a maximum care hospital in lower Saxony with a supraregional catchment area, as well as a FESSH-accredited hand trauma centre, this data cannot be generalised to the country.

Due to the retrospective character of the study, no information about the injury mechanisms is available, thus a proper ICD-10-GM coding by the treating physicians has to be assumed. Previous operations or re-operations in other hospitals were not reported in the current database and therefore cannot be taken into account. As there is no coding for sports injuries available, we utilised an extensive keyword analysis for data collection. Although performed with great care, this approach bears the risk of missing cases of sports-related hand injuries.

These data represent the basic stage within the Translating Research into Injury Prevention Practice framework (TRIPP) [45]. This gradual injury prevention model comprises six stages, leading from injury surveillance up to the implementation of developed prevention measures, and should give researchers support in the successful establishment of injury prevention measures in sports. Given that, a comprehensive monitoring of sports injuries is crucially needed in order to successfully guide and promote injury prevention programs. Supplemental support in this intent can be provided by apps or user friendly computer tools, enabling broadly accessible surveillance participation [28, 29].

The high costs associated with hand and wrist injuries underline the economic importance of maximal safety in sports [23]. However, the financial burden of these injuries can reach great extent at individual level, too. Drop-out rates as well as high direct costs, which might not be covered by insurances, increase the barrier to sports participation among the population [23–25]. While surveillance programs for road injuries or occupational injuries are already well-established, the only available tool for injury monitoring on a national as well as a European level is the EU Injury Database (EU IDB) [24, 46]. However, a little more than half of the EU member states are contributing to this database. The free accessible EU IDB recorded a total of 235 sports-related hand and wrist injuries for Germany in the year 2016 only, suggesting a significant lack of data [46]. Another obstacle is the difficulty of monitoring hospital discharges relating to sports injuries. As hospital statistics are mainly based on the ICD coding, which do not include sports injuries, a reliable monitoring is almost impossible at the moment [24].

## Conclusion

Risk profiles for sports-related hand injuries appear to differ not only in terms of age- and sex, but also geographically. Unlike the typical sports-related hand injury profiles in the United States, cycling, football and equestrian sports were responsible for most sports-related hand injuries in our German study cohort. In order to better assess these risk patterns in Europe and subsequently reduce injury rates and the sequelae, accurate injury surveillance programs are necessary. This could be provided by national as well as European hand trauma registries, presuming a comprehensive registry participation.

## Supplementary Information

**Additional file 1.** List of keywords used for data extraction from the ECRDW. Description of data: None.

## Data Availability

The anonymised datasets used and analysed during the current study are available from the corresponding author on reasonable request.

## Abbreviations

ECRDW: Enterprise Clinical Research Data Warehouse
EU IDB: EU Injury Database
ICD-10-GM: International Statistical Classification of Diseases and Related Health Problems 10th Revision German Modification
LOS: Length of stay
TRIPP: Translating Research into Injury Prevention Practice framework
