# Supplementary material for: Hand injuries in sports – a retrospective analysis of 364 cases
Source: BMC Musculoskelet Disord. 2020 Dec 8;21:826. doi: 10.1186/s12891-020-03807-z (PMC7724715; doi:10.1186/s12891-020-03807-z)
Supplement: Supplementary file 1 — Additional file 1. List of keywords used for data extraction from the ECRDW. Description of data: None. [file 12891_2020_3807_MOESM1_ESM.pdf]

Additional file 1:

Translated keyword list, used for data extraction from the ECRDW.

Aerobic, Acrobatics, Athletics, Badminton, Ballet, Ball sports, Bench press, Baseball, Basketball, Mountain climbing, Biathlon, Bike, Billiard, Boarding, Archery, Bouldering, Bowling, Boxing, Bungee, Capoeira, Cheerleading, Cheer, Cricket, Curling, Dance, Dart, Discus, Hang gliding Unicycle, Ice hockey, Figure skating, Ice skating, Ice stock sport, Bicycle, Parachuting, Fencing, Shuttlecock, Fitness, Flying, American Football, Frisbee, Pentathlon, Football, Artistic gymnastics, Golf, Handball, Hip-Hop, Hockey, Inline, Jogging, Judo, Jitsu, Kayak, Martial arts, Canoe, Karate, Skittles, Kickbox, Kite surfing, Climbing, Strength training, Krav Maga, Shot-put, Kungfu, Lacrosse, Landhockey, Langlauf, Athletics, Maga, Martial Arts, Motocross, Mountainbiking, Nordic Walking, Orienteering, Paddling, Paintball, Parkour, Horse Riding, Pilates, Polo, Quad, Cycling, Rafting, Racing bicycle, Tobogganing, Rollerblading, Rowing, Rugby, Toboggans, Skate, School sports, Swimming, Sailing, Skating, Ski, Skiing, Ski-jumping, Slackline, Snowboard, Snowboarding, Javelin throw, Climbing, Squash, Stand up paddling, Tap dancing, Surf, Surfing, Taek, Taekwondo, Diving, Tennis, Table football, Table tennis, Trampolining, Trekking, Turning, Volleyball, Vaulting, Wake boarding, Wakeboarding, Walking, Hiking, Water ball, Water ski, Yoga, Decathlon, Zumba
